# Prehospital Extremity Fracture Management in Low and Middle‐Income Countries: A Scoping Review of Lay First Responders and Traditional Bonesetters

**DOI:** 10.1002/wjs.12678

**Published:** 2025-07-11

**Authors:** Aayush Unadkat, Emily Stoller, Haleigh Pine, Zachary J. Eisner, Maxwell C. Klapow, Ashwin J. Kulkarni, Anagha Thiagarajan, Nathanael Smith, Peter G. Delaney

**Affiliations:** ^1^ University of Michigan Medical School Ann Arbor Michigan USA; ^2^ LFR International Los Angeles California USA; ^3^ Washington University in St. Louis St. Louis Missouri USA; ^4^ Perelman School of Medicine at the University of Pennsylvania Philadelphia Pennsylvania USA; ^5^ Division of Plastic and Reconstructive Surgery Keck School of Medicine of the University of Southern California Los Angeles California USA; ^6^ Department of Social Policy and Intervention University of Oxford Department of Experimental Psychology Oxford UK; ^7^ Department of Anesthesiology University of Miami Miller School of Medicine Miami Florida USA; ^8^ University California Irvine School of Medicine Irvine California USA; ^9^ Department of Emergency Medicine Washington University School of Medicine St. Louis Missouri USA; ^10^ Cleveland Clinic Department of Orthopaedic Surgery Cleveland Ohio USA

**Keywords:** emergency medical services, fracture management, LMIC, prehospital, splinting

## Abstract

**Purpose:**

Low‐ and middle‐income countries (LMICs) experience the highest rates of injury‐related deaths globally, exacerbated by a lack of robust emergency medical services (EMS). Though fractures contribute substantially to global injury, little is known about prehospital management of extremity fractures in LMICs.

**Methods:**

This review included literature published between January 2000 and January 2024. Inclusion criteria pertained to prehospital settings, defined as care rendered prior to hospital presentation, including care provided by lay first responders (LFRs), professional EMS personnel, and traditional bonesetters (TBS). Multiple authors used the Newcastle‐Ottawa scale to assess texts meeting inclusion criteria, extracting relevant details for analysis.

**Results:**

Of 1251 articles identified, 25 met inclusion criteria. Studies spanned 9 countries across 4 continents, with 14 articles studying care by TBS, 9 by LFRs, and 2 by other prehospital providers. LFR training courses report a combined weighted average pre‐/post‐course difference of 29.16 percentage points. A total of 67% of included studies report adverse outcomes associated with TBS‐managed fractures in the prehospital setting. TBS care is often sought prior to hospital presentation due to sociocultural beliefs, accessibility, and cheaper costs. Few training courses for TBS have been performed, though one course reports a 20.4% increase in fracture management knowledge.

**Conclusion:**

In certain resource‐limited settings, TBS provide most initial fracture management, which may adversely impact outcomes. Knowledge transfer has been demonstrated during prehospital fracture management courses for LFRs and TBS. Early evidence suggests TBS training and integration into healthcare systems may reduce complication rates, improving long‐term outcomes.

## Graphical abstract

Low‐ and middle‐income countries (LMICs) experience the highest rates of injury‐related deaths globally, exacerbated by a lack of robust emergency medical services (EMS). Though fractures contribute substantially to global injury, little is known about prehospital management of extremity fractures in LMICs. This review included literature published between January 2000 and January 2024. Inclusion criteria pertained to prehospital settings, defined as care rendered prior to hospital presentation, including care provided by lay first responders (LFRs), professional EMS personnel, and traditional bonesetters (TBS). Multiple authors used the Newcastle‐Ottawa scale to assess texts meeting inclusion criteria, extracting relevant details for analysis.

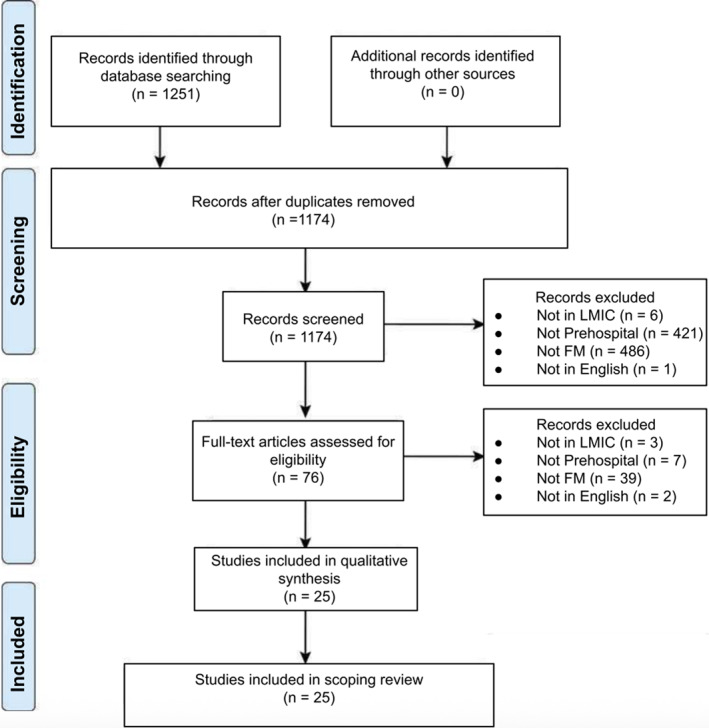

## 1 Introduction

Globally, musculoskeletal injuries disproportionately occur in low‐ and middle‐income countries (LMICs), where 90% of global mortality due to injury is faced [1]. Road traffic incidents (RTIs) are the single greatest contributor to the global injury burden, and with falls, comprise the two leading causes of fracture [2]. Prior to hospital presentation, whether in the emergent or nonemergent setting, LMICs often lack robust prehospital fracture care, and thus patients rarely receive appropriate intervention in this phase such as splinting or intravenous (IV) antibiotics [3]. With fractures heavily implicated as a source of injury‐related disability, early and effective stabilization is recommended to limit deformity, potential soft tissue complications, and minimize pain and morbidity [4, 5]. Prehospital fracture management (FM) is particularly important for open fractures requiring expedient surgical intervention and antibiotic delivery, which otherwise have increased risk of infection and poor outcomes [6].

Over 90% of the population in sub‐Saharan Africa lacks access to formal emergency medical services (EMS) [7, 8]. This deficit in prehospital management can be addressed by training lay first responders (LFRs) in basic first aid and trauma treatment, using curricula emphasizing splinting, minimization of limb movement, and local wound care to mitigate further contamination. Prior to presentation to a hospital, some patients who have sustained fractures may be addressed by EMS or LFRs. However, patients with fractures in LMICs may also utilize traditional bonesetters (TBS), who may attempt closed reduction or splinting acutely (prior to hospital presentation), or as definitive management. These patients often view TBS treatment as a more culturally acceptable and cost‐effective option versus formal orthopedic care, which also may be seen as more likely to result in amputation per qualitative reports [9, 10]. TBS are often trained through familial tradition and apprenticeship, using practices such as herbal mixtures, closed reduction, spiritual incantations, and scarification, with significant heterogeneity existing between providers [11, 12]. Without standardized practices or the use of fluoroscopy, TBS may malreduce fractures, leading to complications including infection, malunion, and amputation [13]. Partly due to patients delaying hospital care to seek TBS treatments, an observational study across 18 low‐income and middle‐income countries found over 70% of open fractures surpass a two‐hour interval from hospital presentation to IV antibiotic delivery [14].

Given the inaccessibility of fracture‐related prehospital care and TBS practices associated with poorer outcomes, formally trained prehospital fracture management training may be beneficial in improving adverse outcomes using socioculturally informed and evidence‐based practices. Literature on prehospital fracture care in LMICs and its relation to TBS remains sparse. This review aims to examine current prehospital training programs for LFRs and professional EMS personnel while contextualizing the role of TBS in LMIC fracture management in order to define the current state of prehospital fracture management and training in LMICs.

## 2 Methods

### 2.1 Literature Search

A scoping review was conducted on prehospital FM in LMICs, which were classified as countries with a gross national income per capita of $12,535 or less, as defined by the World Bank [15]. Utilizing PRISMA‐Scr guidelines [16], the authors reviewed Medline, PubMed, and Scopus databases for articles published between January 1, 2000 and January 1, 2024 using the following search terms: “(splinting OR fracture splinting OR fracture management OR sling OR sling and swath OR wire ladder splint OR traction splint OR extremity fracture OR long bone fracture OR bone immobilization OR fracture immobilization OR fracture stabilization OR SAM splint OR KED Board OR air splint OR vacuum splint) AND (low‐ and middle‐income country OR LMIC OR developing country OR developing countries OR low‐ and middle‐income countries OR low‐income country OR middle‐income country OR Africa) AND (prehospital OR emergency medical services OR EMS OR first responder OR first aid OR injury OR trauma).” Only studies published in English were included with the timeframe being selected to include contemporary research on traditional fracture management strategies and incorporating early studies on bystander intervention since the WHO recommendation in 2005 [17].

**FIGURE 1:**
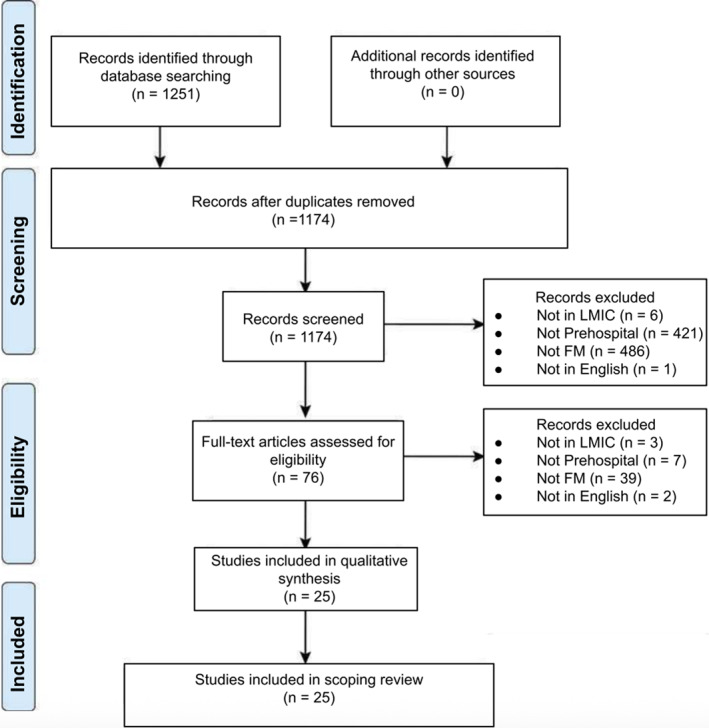
Systematic scoping review protocol.

Studies meeting inclusion criteria pertained to prehospital fracture management in LMICs. Providers included professional EMS personnel, LFRs, or TBS. Professional EMS personnel were defined as formally trained and employed first responders, either part‐ or full‐time, responsible for addressing and transporting patients from the scene to definitive care. LFRs include bystanders not formally employed as first responders, such as law enforcement, transportation providers, and civilians who perform prehospital interventions, which may include transportation to definitive care. TBS describe providers who may or may not have completed formal education or apprenticeships and who use local medicine and manual closed reduction for fracture management. Studies not focusing on fracture management, conducted in high‐income countries, or focusing on hospital settings (nonprehospital) were excluded (Table 1). Prehospital was defined as any care rendered prior to hospital presentation for definitive operative or nonoperative management (Table 1). The review protocol is summarized in Figure 1.

**TABLE 1:** Inclusion and exclusion criteria for scoping review.

	Inclusion criteria	Exclusion criteria
Location	Low‐ and middle‐income countries	High‐income countries
Setting	Prehospital traditional bone setting clinics	Hospital
Clinical scope	Fracture management	Not related to fracture management
Dates	January 1, 2000–January 1, 2024	Before January 1, 2000
Study types	All study types	

### 2.2 Data Collection

Two authors (AU and ES) independently searched Medline, PubMed, and Scopus databases. All manuscripts matching the search keywords were compiled in Microsoft Excel (Microsoft Corporation, Redwood WA) and duplicates were removed electronically before assessing abstracts. Titles, abstracts, and full texts were reviewed independently by two authors prior to inclusion. Discrepancies were resolved via discussion until consensus was reached, with an independent third party (HP) resolving remaining disputes. Both authors (AU and ES) then reviewed manuscripts prior to final inclusion. To compare first responder training programs, this study included manuscripts considering TBS as a prehospital service or described methods and perceptions around their practice. Following manuscript collection, data were extracted independently and compiled for analysis (R Foundation for Statistical Computing, Vienna, Austria). Extracted data included title, authors, publication year, country, LMIC status, level of training, training duration, fracture characteristics, treatment and equipment used, motivations for using TBS, prevalence of adverse outcomes, clinical impact of prehospital intervention, funding source, and participant count. Studies were further distinguished based on the cost of prehospital FM training and TBS methods (Supporting Information S1).

### 2.3 Quality Assessment

Each included study was scored using the Newcastle‐Ottawa scale, which assesses methodological quality in terms of selection, comparability, and outcome [16] (Supporting Information S1). The maximum possible score is 9, with scores of 0–2 considered poor quality, 3–5 considered fair quality, and 6–9 considered high quality. Quality assessment followed an identical protocol as the study selection where two authors scored each manuscript and discrepancies were resolved via discussion and consensus with a third author.

## 3 Results

Initial search generated 1390 articles across PubMed Central, Medline, and Scopus databases, with 1251 studies published between January 1, 2000 and January 1, 2024. Seventy seven articles were excluded as duplicates. Of the 1174 articles remaining, 914 titles did not meet inclusion criteria. Abstracts were then reviewed following established inclusion/exclusion criteria, leaving 76 articles. After reviewing all 76 full‐texts, 51 articles were excluded, with 25 papers included in the study (Figure 1).

### 3.1 Newcastle‐Ottawa Quality Assessment

Of the 25 included studies, 13 (52.0%) utilized cohort, case‐control, or nonrandomized methodologies and were eligible for quality assessment (Table 2). The mean NOS score was 7.40 (median = 8), indicating that studies were of high quality with minimal risk of bias. All included studies had a NOS score of 4 or higher.

**TABLE 2:** Equipment used in TBS practice and during LFR training programs.

	Equipment	% of studies that used element (*n*)
Use by Traditional Bone Setters	Herbal medicine	40.0% (10)
	Makeshift splint using rigid, locally sourced materials bound by soft material (rigid materials including wood, cardboard, bamboo stick, palm stick, rattan cane, plywood, magazine, or tree bark) (soft material including cloth, padding from mattress, folded papers, dried banana leaf, palm leaf, newspaper, and fabric placemat)	32.0% (8)
	Gauze/bandages	12.0% (3)
	Water (either hot and/or potable)	12.0% (3)
	Animal fat/oils/products	8.0% (2)
	Makeshift soft splint (such as cloth or bandage wrapped around the limb without the use of supplementary equipment)	8.0% (2)
	Radiography	8.0% (2)
	Antirotation device	4.0% (1)
	Antiseptic	4.0% (1)
	Leeches	4.0% (1)
	Plaster of Paris	4.0% (1)
	Sterile cotton	4.0% (1)
	Unspecified splint	4.0% (1)
Used by LFRs	Makeshift splints (such as cardboard splints or wooden boards bound by fabric cloth or bandage)	36.0% (9)
Professional EMS personnel	Makeshift splint (such as two pieces of sugar cane, two bungee cords, tape, and a small towel or beanbag splint or cloth/bandage)	8.0% (2)
	Pain medications	4.0% (1)

### 3.2 Study Characteristics

Eighteen of the included studies (72.0%, *n* = 18/25) were conducted in Africa, including Nigeria (*n* = 8), Ghana (*n* = 7), Tanzania (*n* = 1), Rwanda (*n* = 1), or Sierra Leone (*n* = 1). Five studies (*n* = 5/25, 20.0%) were set in Southeast Asia, including India (*n* = 4) and Myanmar (*n* = 1), one (*n* = 1/25, 4.0%) in Central America (Guatemala), and one (*n* = 1/25, 4.0%) in South America (Brazil). Of the manuscripts reviewed, 56.0% (*n* = 14/25) examined TBS practices and reasons for utilization, whereas 36.0% (*n* = 9/25) focused on training and evaluating LFRs. Two studies evaluated training and evaluation of professional EMS providers.

### 3.3 TBS and LFR Training Characteristics

Of 14 TBS studies from Nigeria (*n* = 8), Ghana (*n* = 4), India (*n* = 1), and Tanzania (*n* = 1), 57.1% (*n* = 8) addressed the lack of formal medical training and 28.6% (*n* = 4) evaluated apprenticeship with family members as a method of training, but no information on frequency, cost, or curriculum was available. Two studies (*n* = 2, 8.0%) described training for TBS, one of which indicated a duration of 4 days, and had no mention of refresher training intervals. Five of nine studies pertaining to LFRs detailed prehospital trauma courses for which the median length of training was 6 hours (IQR: 5 h, 4.5 days), but ranged from 5 hours to 1 week. The median cost of LFR training was $5.09 per participant, as reported in three studies. Participants in LFR programs (*n* = 5) consisted of lay person bystanders (*n* = 3), commercial transportation providers (*n* = 3), and law enforcement personnel (*n* = 3). Professional EMS providers include nonphysician health workers, including professional EMS and health workers in mobile clinics.

### 3.4 Equipment and Methods of Care

TBS frequently incorporated additional materials like animal hides, fats, and oils (*n* = 4) and herbal remedies (*n* = 14; Table 2), whereas some urged patients to obtain radiographs from hospitals prior to using TBS services (*n* = 3). Among TBS studies (*n* = 14), many included articles (*n* = 8) reported TBS performing closed reduction maneuvers. By contrast, LFR training predominantly emphasized splint application techniques in situ using local materials (*n* = 8) prior to hospital presentation for definitive management, no manipulation of extremity deformities, application of gauze over grossly open fractures, and transport to definitive care at hospitals (Table 2). Professional EMS personnel had a similar practice scope and additionally employed medications for pain management (*n* = 1; Table 2).

### 3.5 Utilization of TBS Versus Formalized Hospital Care

Across included studies (*n* = 25), twelve—predominantly from West Africa—included estimates on the percentage of fracture patients utilizing TBS care, which ranged from 18.0% to 90.0%, with an IQR of 52%–85% [18, 19, 20, 21, 22, 23, 24, 25, 26, 27, 28, 29]. Three studies included the percentage of patients using both TBS and hospital care, one study estimating 32.6% of patients and the other two estimating 85.0% [26, 28, 29]. Twelve percent of included studies (*n* = 3) noted patients may delay hospital presentation until complications arise following TBS treatment. Time between TBS treatment and hospital presentation varies based on the severity of complications. Severe complications, such as infection and neurovascular compromise, lead patients to present sooner. In two studies in Ghana, 30.4% and 63.0% of patients sometimes left hospitals to utilize TBS care due to convenience, dissatisfaction with care, or financial reasons [22, 26].

Many patients prefer TBS care to hospital care, due to lower costs [18, 21, 22, 30], belief TBS care heals fractures faster [22, 29], increased accessibility [18, 21, 22], sociocultural beliefs [21, 22], and pressure from friends and family [21, 30]. The median cost for adult TBS care was $30.00USD (IQR: $8.98, $172.16), whereas the median cost for hospital‐based FM was $173.31USD (IQR: $98, $268.85) [20, 26, 27, 28, 30]. Some TBS allow flexible payment options, such as monthly installments averaging $1.11USD (*n* = 1), or alternative payment methods, such as accepting livestock as a form of payment (*n* = 3). Additionally, some only request payment upon successful treatment (*n* = 2). The cost of fracture care by TBS varies based on fracture type and location; thus, studies providing a cost range were averaged to determine central tendency.

### 3.6 Outcomes of TBS Care

TBS interventions are associated with numerous complications, which are associated with increased morbidity and mortality. Among two studies (*n* = 2) discussing mortality rates after receiving TBS treatment, one reported a mortality rate of 26.7% over 5 years [12]. Malunion and nonunion were the most prevalent contributors to morbidity, ranging from 16.1% to 64.3% [18, 19, 28, 30, 31]. Additional complications included gangrene (2.8%–29.6%) [19, 30, 31], amputations secondary to gangrene (2.8%–60.0%) [20, 31], limb deformities (2.8%–28%) [19, 30, 31], osteomyelitis (2.8%–3.6%) [30, 31], and Volkmann's ischemic contracture (0.6%–2.8%) [30, 31].

Seven studies evaluated the clinical impact of TBS interventions on preventable complications, including morbidity and mortality. One study from Myanmar indicated that half of patients relied exclusively on TBS, whereas the other half used both TBS and formalized hospital care [31]. Although advantages of TBS care include reduced costs, increased accessibility, and adherence with sociocultural beliefs, hospital care is often sought out for pain and wound management (78.1% of respondents), “complicated” fractures deemed beyond scope of TBS providers (65.6% of respondents), and availability of facilities that can obtain radiographs (27.3% of respondents) [21].

### 3.7 Potential for Integration of TBS Into Health System

As a result of complications that can arise from TBS care, 24% (*n* = 6) of included studies advocated for the integration of TBS into primary healthcare systems, emphasizing the need for formalized education and training courses, an interest shared by TBS in three studies [19, 22, 27]. One study for training TBS reports a 19.7% increase in knowledge (*p* < 0.0001) after completing training modules [19]. In Nigeria, trained TBS have fewer complications than those who are untrained (*p* < 0.05) [19].

Feasibility studies (*n* = 5) demonstrate a combined weighted average pre‐/post‐course difference of 29.16% points (IQR: 11.61, 29.8), measured up to 1‐week post‐course. Most courses focus on general knowledge of fracture management and splinting practices, with trained commercial drivers reporting significantly more confidence in post‐crash care compared to nontrained drivers (*p* = 0.006) [32]. Though fracture management was performed correctly by 97% of a trainee cohort in simulated evaluations 1‐week post‐training and 96% at 1 month follow‐up, there was significant knowledge decay at 13 months (*p* < 0.001) [33]. Additionally, a simulation study in Sierra Leone evaluating LFRs under direct observation by checklist with standardized patient actors determined that fracture immobilization scenarios demonstrated the greatest range in performance by discrete first aid checklist completion (77.8%–97.1%) across scenarios [34].

Given the need for ongoing skill maintenance, continuous quality improvement (CQI) programs have been implemented in hospitals to monitor care delivered by professional EMS providers in Rwanda, demonstrating improved pain management and splinting by 6.1% (*p* = 0.017) and care delivery by 0.7% per month, as measured by the composite trauma quality score measuring the appropriate provision of various prehospital interventions (*p* = 0.028) [35].

## 4 Discussion

Although significant deficits in prehospital FM in LMICs exist, research on formalized FM training remains limited. Of studies meeting inclusion criteria, 12.0% were cohort studies, whereas 40.0% were case‐control studies or case reports. Fourteen studies pertained to TBS, two of which focused on training. Nine examined LFRs, whereas the remaining two assessed other professional first responders, with splinting being the most emphasized curricular element in all trainings. An emphasis on splinting during formal training is needed, as splints applied circumferentially acutely after injury may not permit swelling and may result in ischemia and tissue necrosis [20]. In one study, malunion and nonunion combined accounted for 61.2% of observed complications by patients seen by TBS, with estimates suggesting that 60% of major limb amputations in Nigeria result from TBS‐induced gangrene [36, 37].

Despite outcomes associated with higher morbidity and mortality, patients commonly present to TBS for FM in many LMIC settings. Patient rationale for TBS utilization concerns perceived cost of care, sociocultural beliefs, and fears regarding potential surgery or amputation during hospitalization [12, 18, 21, 22, 24, 25, 26, 27, 29, 30, 38, 39]. Multiple studies found higher costs for formal hospital intervention [21, 26, 37], though one in Lagos, Nigeria found TBS care for closed fractures to range up to $282 USD more expensive than standard care received at a large orthopedic center, potentially secondary to additional episodes of care resulting from complications [30].

Prehospital FM varies significantly across LMIC settings. A study in India found that 31.6% of open fractures were not splinted upon hospital presentation, whereas 48.4% lacked wound dressing [40]. Open fractures significantly increase the risk of hypovolemic shock in cases of polytrauma, further emphasizing the need for rapid and safe management [41]. Existing literature also suggests that even in situations where professional emergency response is deployed, access to definitive care can be obstructed. One study examining 18 LMICs observed that open fracture cases transported by ambulance were delayed at a significantly higher rate as compared to other modes of transport such as rickshaws [14]. Further, FM rendered by untrained bystanders poses unique risks in the prehospital stage itself. In rural Ghana, a survey examining child injuries revealed fractures received the highest percentage (18.0%) of potentially harmful first aid practices, compared to burns, lacerations, or choking, which were described as treating the child within the home only or taking them to a TBS [23]. Given these deficiencies, increased emphasis has been placed on training methodologies that can empower lay responders as well as TBS to stabilize injuries before referral to a physician. In doing so, risks of reinjury, infection, and disability are decreased through the use of evidence‐based practices.

Trainings for LFRs in FM has led to significant knowledge improvements, with post‐course evaluations from LFR‐related studies demonstrating a 29.16% point mean increase in prehospital FM knowledge from pre‐course test scores [33, 42, 43, 44, 45]. LFR training in Sierra Leone demonstrated a 30.4% point increase in FM scores post‐course, with similar results observed on fracture immobilization skills tests in Brazil after fracture care training delivered through seven‐minute videos shown on television. The studies revealed significant knowledge decay at 13 weeks and 9 months, respectively, highlighting the need for follow‐up and refresher training to sustain knowledge [33, 43]. Another study showed benefits of FM training for LFRs compared to other forms of trauma care. After a six‐hour trauma course, LFRs showed the greatest increase in splinting utilization compared to other first aid skills, highlighting effectiveness of knowledge transfer [45]. The aforementioned study from Sierra Leone also highlighted that FM skills were used in 55.6% of patient encounters, second only to hemorrhage control at 61.2% [43]. In the context of variable outcomes observed with TBS practice, these LFR knowledge improvements may indicate a promising route for improved prehospital FM, with a need for further monitoring and evaluation. Although most of the LFR literature base evaluates knowledge acquisition, more recently, a prospective evaluation of the multisite Cameroon Trauma Registry evaluating the association of bystander intervention with reduced early mortality among injury victims found patients presented to hospitals with closed fractures in 22% of all trauma presentations, and only 1 in 5 (21%) had received any immobilization in the prehospital setting. Multivariate logistic regression adjusted for injury severity found that the cohort of patients who had received any prehospital FM at all was associated with increased injury survival [46]. Together, these findings reflect the importance of expanding accessibility to prehospital FM through cost‐effective training.

Integrating and training existing human resources like LFRs and TBS presents an opportunity to scale skilled care more rapidly in resource‐limited settings. In Tanzania, all TBS interviewed expressed willingness to participate in future musculoskeletal training initiatives [27]. Similarly, focus group discussions among orthopedic specialists and TBS in Nigeria revealed mutual interest in establishing pathways for TBS to become trained prehospital technicians who can refer patients [24]. TBS may be integrated into formal orthopedic care networks to receive standardized training, which may subsequently result in fewer complications, as one study resulted in a 48.9% decrease in amputations and a 72.0% decrease in gangrene over 2 years [47]. In Ghana, a 4‐day TBS training program led to a significant increase in splinting knowledge and ability to recognize fracture complications. In addition, this course emphasized irrigating open fractures with potable water to remove gross contamination [19, 48, 49]. Along with post‐test follow‐up at 6 months, researchers found TBS had referred 37 fracture cases to local hospitals via a WhatsApp referral system. Leveraging the prevalence and cultural standing of TBS with effective FM techniques and pathways to refer to orthopedic specialists may improve outcomes in other settings. Further, given the relatively low concentration of orthopedic specialists in LMIC settings compared to high‐income countries, further TBS training and integration (task shifting) may facilitate expanded orthopedic bandwidth and scope by off‐loading nonoperative FM and follow‐up in otherwise constrained environments where TBS care is prevalent while reducing complication rates and improving long‐term TBS outcomes.

Overall, literature examining the status of prehospital FM in LMICs is limited. Additional limitations to this review include restricting articles to those written in English and indexed in PMC, MEDLINE, and Scopus databases. Although broad generalizations should not be drawn from a review of 25 articles spanning five sub‐Saharan countries and four other LMICs, consistency of adverse outcomes from TBS care and significant knowledge transfer during short LFR trauma courses is likely suggestive of the current experience in alternative LMIC environments. These findings underscore a need for further research and investment in prehospital LFR training, and highlight the potential for TBS integration into formal care networks to alleviate the burden of disability and mortality associated with limited FM resources in LMICs.

## 5 Conclusion

Prehospital fracture management is performed by various providers across LMICs. In settings where TBS are common, care is associated with complications, which may be exacerbated by a lack of formal medical education. Training TBS and LFRs is an economically feasible and effective way to improve prehospital fracture management, which has been associated with improved outcomes in limited studies. TBS integration into formal healthcare systems may lead to early complication recognition and patient referral while recognizing the value of TBS in a socioculturally‐informed manner.

## Author Contributions

**Aayush Unadkat:** conceptualization, investigation, writing – original draft, methodology, writing – review and editing, formal analysis, project administration, data curation, validation, visualization. **Emily Stoller:** conceptualization, investigation, writing – original draft, methodology, validation, writing – review and editing, formal analysis, project administration, data curation, software. **Haleigh Pine:** investigation, writing – original draft, writing – review and editing, supervision, project administration, formal analysis, conceptualization. **Zachary J. Eisner:** validation, project administration, supervision, writing – review and editing. **Maxwell C. Klapow:** validation, project administration, supervision, writing – review and editing, formal analysis. **Ashwin J. Kulkarni:** formal analysis, investigation, methodology. **Anagha Thiagarajan:** investigation, writing – original draft, validation. **Nathanael Smith:** writing – review and editing, supervision, project administration. **Peter G. Delaney:** supervision, conceptualization, writing – original draft, writing – review and editing, validation, methodology, project administration, resources.

## Ethics Statement

The authors have nothing to report.

## Conflicts of Interest

The authors declare no conflicts of interest.

## Supporting information

Table S2

## Data Availability

Data sharing not applicable to this article as no datasets were generated or analyzed during the current study.
